# TMT-based comprehensive proteomic profiling identifies serum prognostic signatures of acute myeloid leukemia

**DOI:** 10.1515/med-2022-0602

**Published:** 2023-03-30

**Authors:** Wei Zhang, Bei Liu, Shiwen Wu, Li Zhao

**Affiliations:** Department of Central Laboratory, The First Hospital of Lanzhou University, Lanzhou 730000, Gansu Province, China; Department of Hematology, The First Hospital of Lanzhou University, Lanzhou 730000, China; Department of Laboratory Medicine, The First Hospital of Lanzhou University, Lanzhou 730000, China; Department of Central Laboratory, The First Hospital of Lanzhou University, #1 Donggang West Road, Lanzhou 730000, Gansu Province, China

**Keywords:** TMT labeling-based quantitative proteomics, isocitrate dehydrogenase 2, favorable-risk acute myeloid leukemia, prognostic biomarker, proteomic profiling

## Abstract

Acute myeloid leukemia (AML) is classified into favorable-risk, intermediate-risk, and poor-risk subtypes. This study aimed to compare the serum proteomic signatures of the three AML subtypes and identify prognostic biomarkers for AML. Serum samples from patients with favorable-risk (*n* = 14), intermediate-risk (*n* = 19), and poor-risk AMLs (*n* = 18) were used for the analysis of tandem mass tag (TMT) labeling-based quantitative proteomics. Comparative analysis was performed to identify differentially expressed proteins (DEPs) between groups. Prognostic proteins were screened using binary logistics regression analysis. TMT-MS/MS proteomics analysis identified 138 DEPs. Fumarate hydratase (FH), isocitrate dehydrogenase 2 (IDH2), and enolase 1 (ENO1) were significantly upregulated in poor-risk patients compared with favorable-risk patients. ELISA assay confirmed that patients with poor-risk AMLs had higher levels of IDH2, ENO1, and FH compared with intermediate-risk AML patients. Logistics analysis identified that proteins 3-hydroxyacyl-CoA dehydrogenase type-2 (HADH, odds ratio (OR) = 1.035, *p* = 0.010), glutamine synthetase (GLUL, OR = 1.022, *p* = 0.039), and lactotransferrin (LTF, OR = 1.1224, *p* = 0.016) were associated with poor prognosis, and proteins ENO1 (OR = 1.154, *p* = 0.053), FH (OR = 1.043, *p* = 0.059), and IDH2 (OR = 3.350, *p* = 0.055) were associated with AML prognosis. This study showed that AML patients had elevated levels of FH, IDH2, ENO1, LTF, and GLUL proteins and might be at high risk of poor prognosis.

## 1 Introduction

Acute myeloid leukemia (AML) is a malignant tumor and the most common acute leukemia in adults [1]. AML has high heterogeneity and great differences in genetic mutations, treatment responses, and prognosis although patients could be classified into favorable-risk, intermediate-risk, and poor-risk subtypes according to the cytogenetic profile [2,3,4]. The prognostic importance of cytogenetic aberrations has been widely accepted over the past 15 years. However, its translation into therapy is just beginning, especially for poor-risk AML patients.

Advance in mining the diagnostic or prognostic biomarkers for AML has been underway for decades. The pandemic and development of techniques in microarray, DNA sequencing, and high-throughput genome sequencing show that there is a significant difference in AML heterogeneity [5,6]. Molecular testing is a major diagnostic work-up for AML and is necessary for classifying subtypes, predicting prognosis, and making treatment decisions [4,7,8]. For instance, AMLs with t(8;21)(q22;q22.1), biallelic mutated *CEBPA*, mutated *NPM1* without *FLT3*-internal tandem duplication (ITD, *FLT3*-*ITD*), rearrangement in inv(16)(p13.1q22) or t(16;16)(p13.1;q22) were favorable-risk, and AMLs with complex karyotype, t(6;9)(p23;q34.1), mutated *TP53*, mutated *ASXL1*, mutated *RUNX1*, and *FLT3*-*ITD* ^high^ are poor-risk [4]. Patients with favorable-risk AMLs have a generally improved overall survival in comparison with patients with poor-risk AMLs [4,9]. The identification of mutations and new molecular markers that carry prognostic impact in AML is increasing with the use of molecular profiling [9].

There is increasing evidence showing that proteomic analysis can help to form therapeutic schedules and provide potent prognostic biomarkers [10,11]. Genetic abnormalities in AML patients have been related to abnormal proteome and metabolome profiles [12]. Also, the functional proteomic profile of AML might predict prognosis or treatment responses [13,14,15,16]. Kadia et al. showed that patients with *RAS* ^mut^ AMLs had elevated expression of RAS-Raf-MAP kinase and phosphoinositide 3-kinase (PI3K) [12]. They showed that RAS mutations may delineate a subset of AML patients who benefit from cytarabine-based therapy and who may be amenable to treatment with inhibitors of RAS and PI3K signaling pathways. Luczak et al. [16] showed that AML-M2-T0 patients who had detectable expression levels of Annexin III, L-plastin, and 6-phosphogluconate dehydrogenase in blood were resistant to treatment. However, there is less information on the comprehensive proteomic profiling of patients with favorable-risk, intermediate-risk, and poor-risk AMLs. Also, biomarkers that have a prognostic impact on AML are badly needed to improve risk stratification [17].

The objective of this study was to identify and compare the serum proteomic signatures of patients with favorable-risk, intermediate-risk, and poor-risk AMLs. In this study, the proteomic landscape and potent serum prognostic biomarkers for AML, irrespective of cytogenetics, were analyzed.

## 2 Materials and methods

### 2.1 Subjects and grouping

Fifty-one AML patients were enrolled from the Department of Hematology, The First Affiliated Hospital of Lanzhou University, Lanzhou, China, between March 2019 and June 2020. All patients were diagnosed and introduced to induction standard chemotherapy according to the National Comprehensive Cancer Network guidelines (2016 edition) [4]. The inclusion criteria for patients were: newly diagnosed AMLs and fit to receive chemotherapy. The exclusion criteria were: acute promyelocytic leukemia (APL, M3 subtype), patients who lacked the information of gene mutation, fusion, or karyotype, and patients with secondary/relapsed AMLs or unfit to receive chemotherapy. There was no restriction on age and gender. Patients were categorized as favorable-risk (*n* = 14), intermediate-risk (*n* = 19), and poor-risk AMLs (*n* = 18) according to the cytogenetic categories [4]. Favorable cytogenetics were defined as the presence of t(8;21), inv(16)/t(l6;16), and t(15;17). Poor cytogenetics were inv(3)/t(3;3), t(6;9), t(9;22), t(v;11q23.3), -17/abn(17p), 17, and -5/del(5q). The AMLs with t(9:11) and cytogenetic abnormalities not classified as favorable or poor were intermediate-risk AMLs. The age, gender, bone marrow (BM) blast percentage, mutation, white blood cell (WBC) count, karyotype, and fusion gene were collected. Gene fusion and mutation of genes (fusion: *BCR-ABL1*, *CBFB*-*MYH11*, *RUNX1*-*RUNX1T1*, *AML1*-*ETO*, *PML*-*RARA*, *ETV6-RUNX1*, *MLL*-*AF4*/6, *MLLT3*-*KMT2A*, *DEK*-*NUP214*, and *PML*-*RARA*; Mutation: *CEBPA* (biallelic), *NPM1*, *FLT3*-ITD, *MLL*-PTD, *RUNX1*, *ASLXL1*, *TP53*, *TET2*, *NRAS*, *C*-*kit*/*D816*, *DNMT3A*, *IDH2*/*R172*, *KIT*, *IDH2*, *WT1*, and *NOTCH1*) were detected using the nested-PCR combined with Sanger sequencing. The BM blast percentage was detected using a BM cell morphological image analysis system (Jieda Technology Development Co., Ltd, Jiangsu, China). The WBC count was examined using a blood cell counter (Mindray BC-6800, Mindray, Shenzhen, China). The karyotype was analyzed using the CytoVision Image Analysis (Leica Biosystems Newcastle Ltd, UK). Serum samples were collected from all participants and used for the tandem mass tag (TMT)-based quantitative proteomics analysis.

#### 2.1.1 Sample preparation, trypsin digestion, and iTRAQ labeling

The cellular debris was removed from serum samples by centrifugation at 12,000*g*, 4°C for 10 min. The supernatant was collected and treated using the Pierce™ Top 12 Abundant Protein Depletion Spin Columns Kit (Thermo Fisher Scientific, IL, USA) to remove the 12 high-abundance proteins. Protein quantification was performed using a BCA kit (Beyotime Institute of Biotechnology, Hangzhou, China) according to the manufacturer’s instructions. Before digestion, the protein solution was reduced with dithiothreitol (5 mM, at 56°C for 30 min) and alkylated with iodoacetamide (11 mM, at room temperature for 15 min, in darkness). Then, the solutions were diluted with tetraethyl ammonium bromide (TEAB; 100 mM) to urea concentration less than 2 M. Finally, proteins were digested using two-step digestion with trypsin (Promega, Madison, WI, USA), 1:50 trypsin-to-protein mass ratio overnight and 1:100 trypsin-to-protein mass ratio for 4 h. For TMT labeling, digested peptides were desalted using a Strata X C18 SPE column (Phenomenex, Torrance, CA, USA), vacuum-dried, dissolved in TEAB, and then prepared using labeling reagents for 2 h at room temperature according to instructions provided by a TMT kit (Thermo Fisher Scientific). Samples were dried by vacuum centrifugation.

#### 2.1.2 High-performance liquid chromatography (HPLC) and tandem mass spectrometry (MS/MS) analysis

The fractionation of tryptic peptides was performed using high pH reverse-phase HPLC and Agilent 300 Extend C18 column (5 μm particles, 4.6 mm ID, 250 mm length; Phenomenex) and was separated with a gradient of 8–32% acetonitrile over 60 min. Peptides were separated into 60 fractions and were then combined into 18 fractions. After drying by vacuum centrifugation, peptides were dissolved in 0.1% formic acid (solution A) and trapped onto a home-made reverse-phase analytical column (15 cm length, 75 μm inner diameter; Phenomenex). The elution was performed on an EASY-nLC 1000 UPLC system (Thermo Fisher Scientific) in a gradient of 9–25% solvent B (0.1% formic acid in 98% acetonitrile) over 26 min, 26–34% over 8 min, 25–38% over 3 min, 38–80% over 3 min, and holding at 80% for 3 min, at a constant flow rate of 700 nL/min. For LC-MS/MS analysis, peptides were subjected to nanospray ionization (NSI) source followed by MS/MS in an Orbitrap Fusion^TM^ Lumos Tribrid mass spectrometer (Thermo Fisher Scientific; 2.0 kV). The scan range of full mode (MS 1) was 350–1,550 *m/z* at a resolution of 6,000, and in MS 2 mode, scanning was started at 100 *m/z* at a resolution of 30,000. Other parameters were: AGC target 5E4, dynamic exclusion 30 s, and maximum injection time 200 ms.

#### 2.1.3 Database search and protein identification

Raw MS/MS data were analyzed using the Maxquant search engine (v.1.5.2.8). Tandem mass spectra were searched against the Swiss-Prot Human database (20,317 sequences; http://www.uniprot.org) concatenated with a reverse decoy database. The parameters were set as follows: trypsin/P was specified as cleavage enzyme, 2 missed cleavages were allowed, the minimum length of peptides was 7, mass tolerance for precursor ions was 20 ppm in the first search and 5 ppm in the main search, and mass tolerance for fragment ions was 0.02 Da, the fixed modification was Carbamidomethyl, variable modification was used as oxidation on Met, fold discovery rate was adjusted to <1%, and the minimum score for peptides was set >40. The differentially expressed proteins (DEPs) between groups were identified based on the requirements of *p* value <0.05 and fold change (FC) ≥1.20 (upregulation) or <0.75 (downregulation). The online Venn diagrams generator BioVenn (https://www.biovenn.nl/index.php) was used to create Venn diagrams of DEPs by different comparisons.

#### 2.1.4 Bioinformatics analysis

The properties and classifications of DEPs were annotated in the Gene Ontology (GO, http://www.geneontology.org) and Kyoto Encyclopedia of Genes and Genomes (KEGG, http://www.genome.jp/kegg/) databases. In addition, functional categories associated with DEPs were identified using the online Database for Annotation, Visualization, and Integrated Discovery (DAVID, v6.8; https://david.ncifcrf.gov/). The interactions between proteins were identified in the STRING database (https://string-db.org/cgi/input.pl). The protein–protein interaction (PPI) network was visualized using Cytoscape (v 3.8.0; http://apps.cytoscape.org/). Significant modules in the PPI network were identified using the MCODE plugin (http://apps.cytoscape.org/apps/mcode) with the threshold of module score ≥5.0.


**Ethical approval:** An approval (LDYYLL2019-72) was obtained from the ethics committee of The First Affiliated Hospital of Lanzhou University, Lanzhou, China. Consent to participate was obtained from each individual.

### 2.2 Enzyme-linked immunosorbent assay (ELISA) assay

A validation cohort of 33 patients with AMLs (5 in favorable-risk, 17 in intermediate-risk, and 11 in poor-risk) and 10 patients with myelodysplastic syndromes, lupus nephritis, or thrombopenia (Control) was used for the validation of ELISA assay. The AML patients were recruited according to the above inclusion and exclusion criteria between June and August 2019. The exclusion criterion for the control patients was patients with cancers and other diseases like sepsis, chronic metabolic syndromes, and cardiovascular diseases. An approval (LDYYLL2019-80) was obtained from the ethics committee of The First Affiliated Hospital of Lanzhou University, Lanzhou, China. Consent to participate was obtained from each individual.

The serum concentration of six potent prognostic DEPs, including enolase 1 (ENO1; Cusabio, Wuhan, China; ng/mL), fumarate hydratase (FH), mitochondrial (Cusabio; ng/mL), glutamine synthetase (GLUL; Abnova, Taipei, China; ng/mL), 3-hydroxyacyl-CoA dehydrogenase type-2 (HADH; Cusabio; pg/mL), isocitrate dehydrogenase 2 (IDH2; Jianglai, Shanghai, China; ng/mL), and lactotransferrin (LTF; Cusabio; μg/mL), was determined using commercial ELISA kits according to instructions. Absorbance at 450 nm was recorded and proteins’ concentrations were calculated using standard curves.

#### 2.2.1 Gene expression profiling interactive analysis (GEPIA) survival verification

The association of potent prognostic protein-encoding genes with AML prognosis was analyzed using the GEPIA online tool (http://gepia.cancer-pku.cn/). This web server is a web-based tool to deliver fast and customizable functionalities based on The Cancer Genome Atlas (TCGA) data. Kaplan–Meier survival curves were generated and downloaded. GEPIA performs overall survival (OS) analysis based on gene expression and divides samples into high and low groups according to the median value of gene expression. GEPIA uses the Log-rank test for the hypothesis test. The Cox proportional hazard ratio (HR) and the 95% confidence interval (CI) were also included in the survival plot.

### 2.3 Statistical analysis

Data were presented as mean value ± standard deviation (SD). All data were statistically analyzed using SPSS 22.0 (IBM, Chicago, IL, USA). The non-parametric Kruskal–Wallis *H* test (Dunn’s test) was used to analyze the differences across groups. Counting variable was expressed as frequency and analyzed using the Fisher’s test. Variables associated with the survival outcomes of patients with AML were identified using univariate and multivariate logistics regression analysis. Differences were considered statistically significant at the threshold of *p* value < 0.05.

## 3 Results

### 3.1 Characteristics of the patients used for TMT-MS/MS analysis

There was no difference in patients’ age, gender ratio, WBC count, and BM blast percent among patients with poor-risk, intermediate-risk, and favorable-risk AMLs (Table 1). Most patients had AML-M5 (*n* = 14, 27.45%), AML-M4 (*n* = 13, 25.49%), and AML-M2 (19.61%, Table 1). Patients with favorable-risk AMLs had mutated *WT1*, *NPM1*, *IDH2* ^R14*0*^, biallelic mutated *CEBPA*, and *TET2*, without *FLT3*-ITD (Table S1); patients with poor-risk AMLs had mutated *FLT3*-ITD, *CEBPA*, and *ASXL1*; and patients with intermediate-risk AMLs had mutated *TET2*, *ASXL1*, *DNMT3A*, *IDH2* ^R172^, and *ASXL1* (Table S1).

**Table 1:** Characteristics of patients with acute myeloid leukemia used for the analysis of TMT-MS/MS and ELISA

Variables	TMT-MS/MS cohort (*n* = 51)			
	Poor-risk (*n* = 18)	Intermediate-risk (*n* = 19)	Favorable-risk (*n* = 14)	*p* value
Age (year)	50.00 (12.00–71.00)	50.00 (10.0–66.00)	47.00 (10.00–78.00)	0.708
Gender (male/female)	11/7	8/11	8/6	0.487^‡^
WBC (10^9^/L)	28.57 (0.67–337.28)	11.62 (0.33–189.37)	15.12 (0.69–123.00)	0.526
BM blast percent (%)	79.50 (29.50–94.00)	80.50 (52.50–94.50)	75.75(49.52–98.50)	0.307

	ELISA cohort (*n* = 43)				
	Poor-risk (*n* = 11)	Intermediate-risk (*n* = 17)	Favorable-risk (*n* = 5)	Control (*n* = 10)	*p* value
Age	47.00 (15.00–82.00)	46.00 (10.00–72.00)	47.00 (35.00–64.00)	48.00 (15.00–72.00)	0.822 (0.649^Δ^)
Gender (male/female)	7/4	6/11	2/3	6/4	0.964^‡^ (0.244^Δ^)
WBC (10^9^/L)	31.05 (2.40–337.28)	12.93 (1.37–131.58)	10.53 (2.34–43.30)	5.51 (2.91–7.56)	0.133 (0.618^Δ^)
BM blast percent (%)	65.00 (42.50–95.00)	75.00 (50.00–94.50)	58.75 (44.00–87.50)	3.50 (2.50–9.40)	0.005 (0.618^Δ^)

BM, bone marrow. ELISA, enzyme-linked immunosorbent assay. TMT-MS/MS, tandem mass tag-based tandem mass spectrometry. WBC, white blood cells. The ‡ symbol indicates that the *p* value of this analysis is analyzed by the Chi-square test, and that all other analyses are performed with the non-parametric Kruskal–Wallis *H* test (Dunn’s test). The Δ symbol denotes the *p* value for the comparison across the first three groups without control.

#### 3.1.1 Analysis of TMT-MS/MS proteomics and DEPs for patients with AML

**Figure 1:**
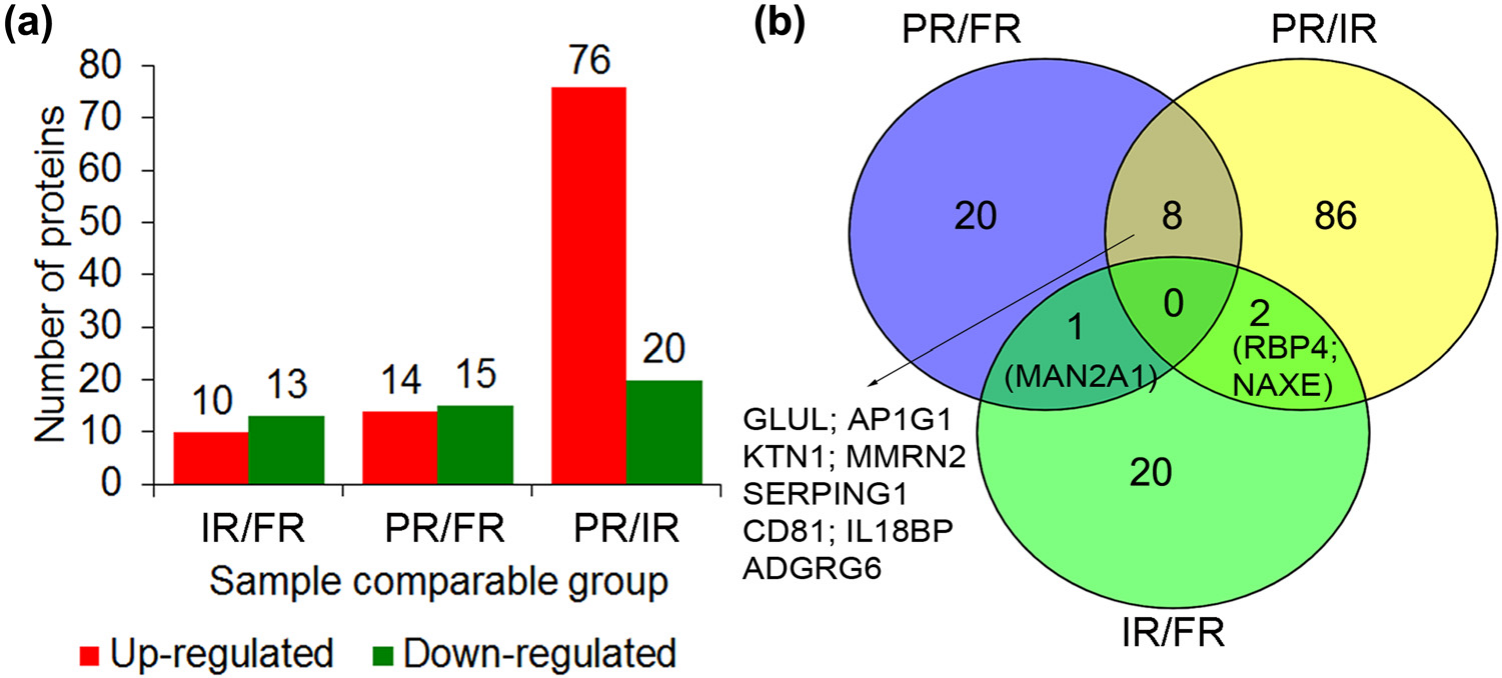
The statistics of DEPs in patients with acute myeloid leukemia. (a and b) The statistics diagram and the Venn diagram of the DEPs by different comparisons, respectively. FR, favorable-risk. IR, intermediate-risk. PR, poor-risk.

TMT-MS/MS proteomic analysis identified that there were 29, 86, and 23 DEPs for the comparisons of poor-risk vs favorable-risk, poor-risk vs intermediate-risk, and intermediate-risk vs favorable-risk, respectively, including 138 non-overlapping DEPs and 8 common DEPs (GLUL; AP-1 complex subunit gamma-1, AP1G1; Kinectin, KTN1; Multimerin-2, MMRN2; serine/cysteine proteinase inhibitor clade G member 1, SERPING1; CD81 antigen, CD81; Interleukin-18-binding protein, IL18BP; Adhesion G-protein coupled receptor G6, ADGRG6; Figure 1a and b). All DEPs are listed in Table S2.

#### 3.1.2 Functional enrichment analysis for DEPs

Functional enrichment analysis showed that DEPs for poor-risk vs favorable-risk were associated with six biological process terms, including “GO:0050829: defense response to Gram-negative bacterium,” “GO:0072675: osteoclast fusion,” and “GO:0045087: innate immune response”; three molecular function terms, including “GO:1990459: transferrin receptor binding,” “GO:0043236: laminin binding,” and “GO:0001530: lipopolysaccharide binding”; and six cellular component terms, including “GO:0070062: extracellular exosome,” “GO:0035580: specific granule lumen,” and “GO:0009986: cell surface” (Table S3).

The DEPs for the comparison of poor-risk vs intermediate-risk were associated with 27 biological process terms, including “GO:0002181: cytoplasmic translation,” “GO:0006397: mRNA processing,” and “GO:0006888: ER to Golgi vesicle-mediated transport”; ten molecular function terms related to the binding of protein, RNA, and cadherin; and 27 cellular component terms involved in proteasome, membrane, nucleus, ribosome, and cell body. The DEPs were associated with four KEGG pathways, including “hsa03010: Ribosome,” “hsa01230: Biosynthesis of amino acids,” “hsa05171: Coronavirus disease-COVID-19,” and “hsa03050: Proteasome” (Table S3).

Besides, DEPs for the comparison of favorable-risk vs intermediate-risk were associated with two biological process terms, including “GO:0006869: lipid transport” and “GO:0010874: regulation of cholesterol efflux”; two molecular function terms, including “GO:0030246: carbohydrate binding” and “GO:0070492: oligosaccharide binding”; and six cellular component terms, including “GO:0070062: extracellular exosome,” “GO:0005615: extracellular space,” and “GO:0005801: cis-Golgi network.”

The results of functional enrichment analysis showed that DEPs were associated with the development of AML by regulating multiple and various biological processes and pathways.

#### 3.1.3 PPI network analysis

**Figure 2:**
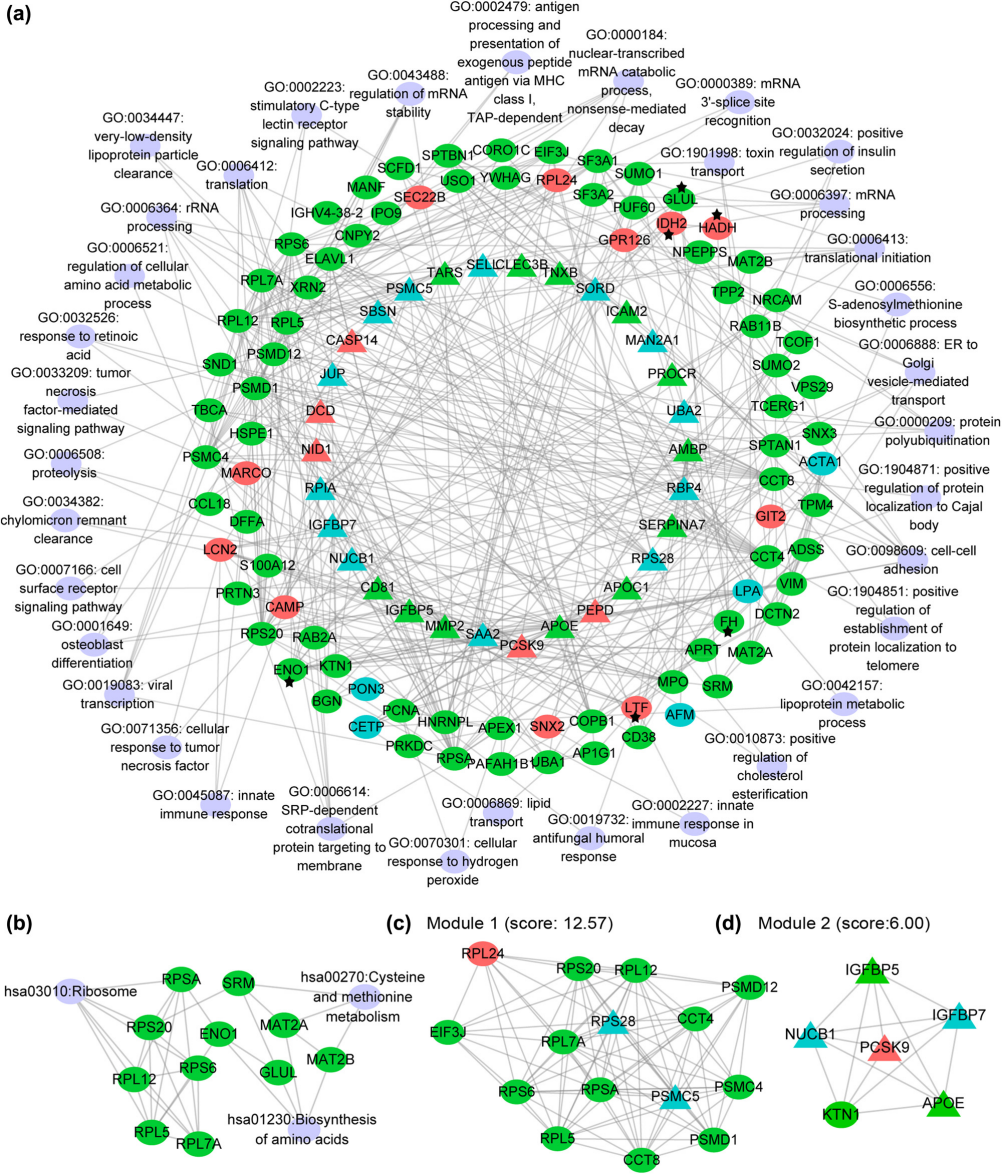
The PPI network of DEPs in patients with acute myeloid leukemia. (a and b) The PPI network of DEPs by different comparisons: favorable-risk vs intermediate-risk (Cyan), favorable-risk vs poor-risk (Red), and intermediate-risk vs poor-risk (Green), involving biological processes and pathways, respectively. Black stars indicate six potent proteins: FH, GLUL, LTF, ENO1, HADH, and IDH2. (c and d) The two modules. Upregulated and downregulated proteins are shown by circles and triangles, respectively. The two significant modules were identified using the MCODE plugin in Cytoscape (http://apps.cytoscape.org/apps/mcode) with the threshold score ≥5.0.

The PPI network of DEPs is shown in Figure 2, in which 114 DEPs (nodes) and 358 interactions (edges) are included. Also, 35 GO biological processes (Figure 2a), three KEGG pathways (Figure 2b), and 42 cellular component terms (Figure S1) were included in the network. Proteins like 40S ribosomal protein S20 (RPS20), 40S ribosomal protein SA (RPSA), ENO1, T-complex protein 1 subunit theta (CCT4), FH, LTF, and IDH2 had interaction degrees of 14, 18, 10, 20, 9, 9, and 6, respectively (Figure 2). Also, HADH had a low interaction degree of two, with IDH2 and FH, in the PPI network. ENO1 interacted with FH, IDH2, CCT4, RPSA, and GLUL (Figure 2a). Following the threshold of score ≥5.0, there are two modules with scores of 12.57 and scores of 6.00. Module 1, 15 nodes and 88 edges, (Figure 2c) and module 2, six nodes and 15 edges (Figure 2d). Module 1 consisted of 14 DEPs by poor-risk vs intermediate-risk, including 26S proteasome non-ATPase regulatory subunit 1 (PSMD1); PSMD12, PSMC4, CCT8, RPSA, RPS6, RPS20, and 60S ribosomal protein L5 (RPL5). These proteins were associated with biological processes including “GO:0006413: translational initiation,” “GO:0019083: viral transcription,” “GO:0006364: rRNA processing,” “GO:0033209: TNF-mediated signaling pathway,” and “GO:0002223: stimulatory C-type lectin receptor signaling pathway” (Table S3 and Figure 2).

#### 3.1.4 ELISA assay of AML-related proteins

**Figure 3:**
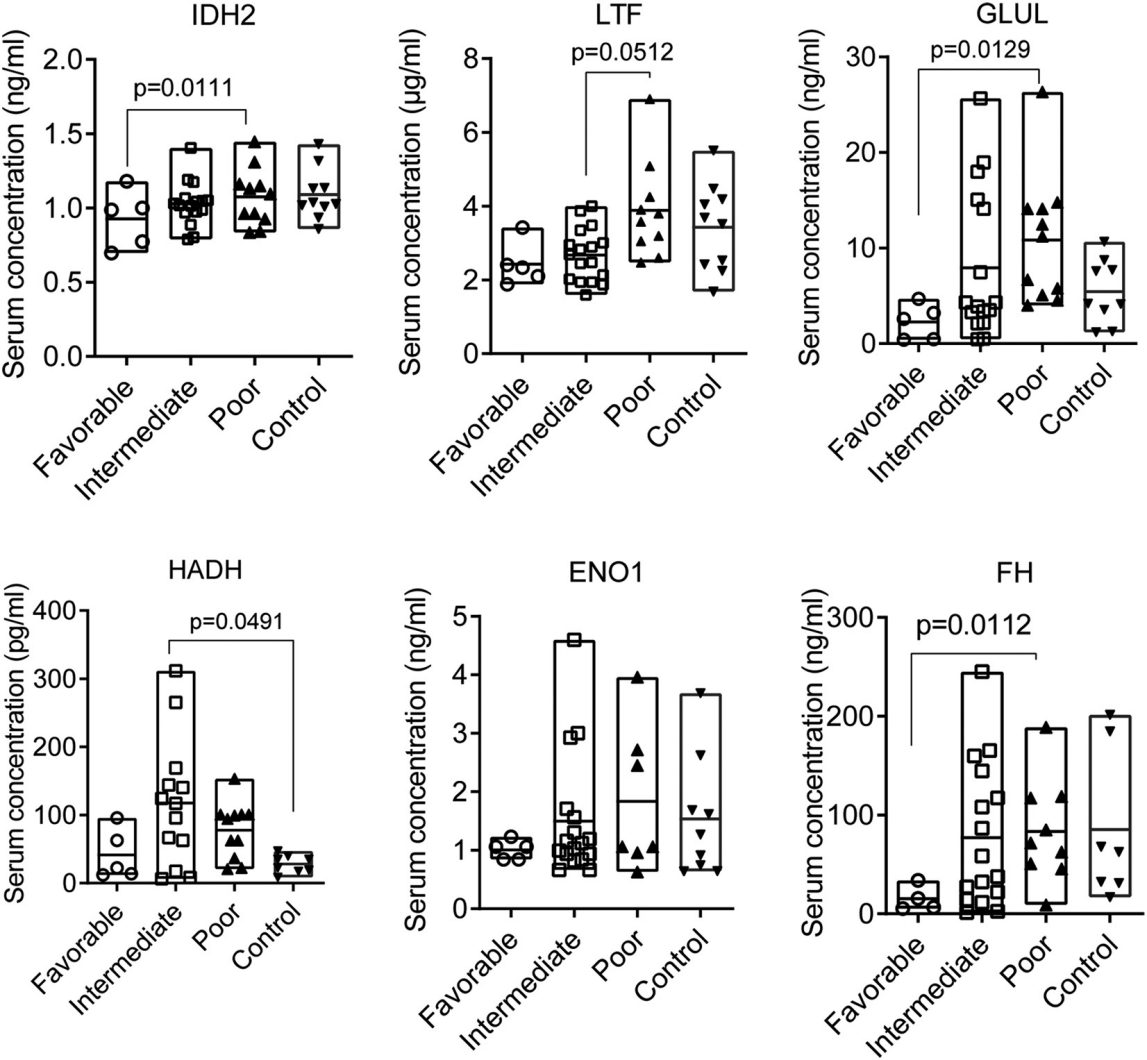
The serum contents of six proteins by ELISA assay. ENO1, enolase 1; FH, fumarate hydratase, mitochondrial; GLUL, glutamine synthetase; HADH, hydroxyacyl-CoA dehydrogenase; IDH2, isocitrate dehydrogenase 2; LTF, lactotransferrin. Differences across groups were analyzed using the non-parametric Kruskal–Wallis *H* test (Tukey *post-hoc* test). Patients with myelodysplastic syndromes, lupus nephritis, or thrombopenia were enrolled as controls. Data are expressed as scattered plots, and the boxed values indicate median with range (min to max).

Among the DEPs, six proteins that had been reported to be related to the prognosis of AML and other cancers and had relative higher FC ratios by comparison (Table S2), including FH (poor-risk vs intermediate-risk, FC = 1.36) [18], GLUL (poor-risk vs favorable-risk, FC = 2.06) [19], LTF (poor-risk vs favorable-risk, FC = 3.15) [20], ENO1 (poor-risk vs intermediate-risk, FC = 1.29) [21], HADH (poor-risk vs favorable-risk, FC = 1.74) [22], and IDH2 (poor-risk vs favorable-risk, FC = 2.25) [18,23], were selected as potent candidates for validation. The ELISA assay confirmed that the serum levels of FH, LTF, ENO1, HADH, and IDH2 proteins were differentially expressed in patients with intermediate-risk, favorable-risk, and poor-risk AMLs (Figure 3). Patients with poor-risk AMLs had higher serum contents of IDH2 (*p* = 0.0177), ENO1 (*p* = 0.0343), and FH (*p* = 0.0080) compared with patients with favorable-risk AMLs (Figure 3). The other proteins had insignificant elevations in patients with intermediate-risk and poor-risk AMLs compared with patients who had favorable-risk AMLs.

#### 3.1.5 Association with prognosis

Binary logistics analysis showed that three of the six proteins, including HADH, GLUL, and LTF, were associated with the survival outcomes in patients with AMLs (HADH: odds ratio (OR) = 1.035, 95% CI 1.010–1.065, *p* = 0.010; GLUL: OR = 1.022; 95% CI 1.003–1.045, *p* = 0.039; and LTF: OR = 1.124, 95% CI 1.255–2.585, *p* = 0.016; Table 2). Logistics analysis showed that AML patients who had higher serum levels of HADH, GLUL, and LTF were at a high risk of poor prognosis. Also, we found that patients with high levels of IDH2 (OR = 3.350, 95% CI 0.988–4.722, *p* = 0.055), ENO1 (OR = 1.154, 95% CI 1.059–4.223, *p* = 0.053), and FH (OR = 1.043, 95% CI 0.998–1.044, *p* = 0.059) were at higher risk of poor prognosis compared with patients with low levels of these proteins (Table 2).

**Table 2:** Variables associated with the survival outcomes of acute myeloid leukemia using univariate and multivariate logistics regression analysis

Variables	Univariate				Multivariate			
	*β*	OR	95% CI	*p*	*β*	OR	95% CI	*p*
IDH2	0.608	5.000	1.032–7.722	0.046	0.638	3.350	0.988–4.722	0.055
HADH	0.033	1.033	1.008–1.058	0.008	0.035	1.035	1.010–1.065	0.010
GLUL	0.176	1.214	1.013–1.455	0.036	0.274	1.022	1.003–1.045	0.039
ENO1	0.574	1.428	1.070–3.043	0.016	0.667	1.154	1.059–4.223	0.053
FH	0.022	1.022	1.001–1.044	0.038	0.043	1.043	0.998–1.044	0.059
LTF	0.650	1.512	1.223–2.135	0.009	0.627	1.124	1.255–2.585	0.016
WBC count	0.027	1.028	0.987–1.070	0.186				
Age	0.002	1.002	0.959–1.047	0.930				
Gender	0.143	1.154	0.255–5.223	0.853				

95% CI, 95% confident interval. OR, odds ratio.

**Figure 4:**
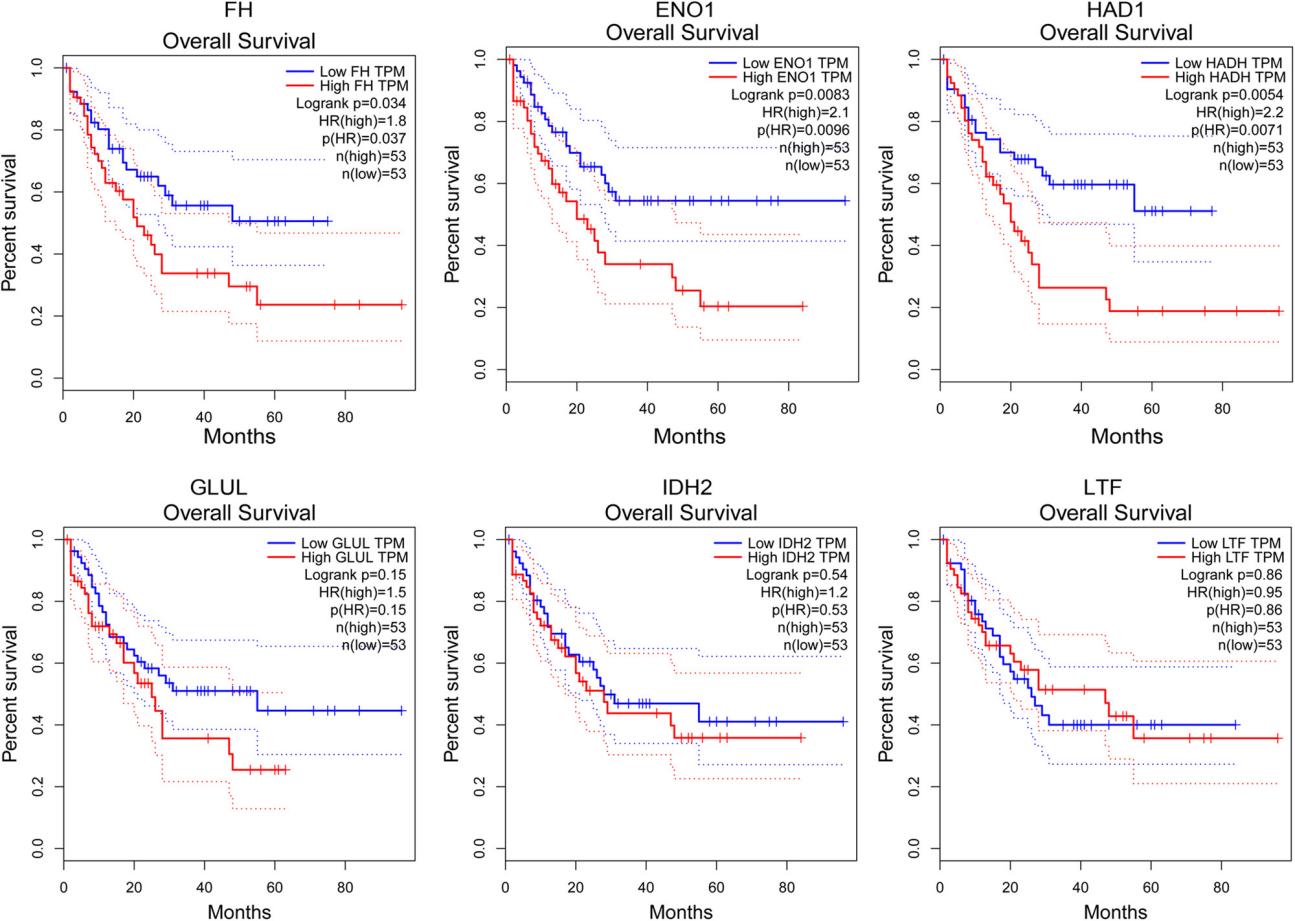
Correlation of protein-encoding genes with overall survival in acute myeloid leukemia. Prognosis verification was performed using the GEPIA online tool, based on the TCGA data (*n* = 106). The cutoff of high and low expression is the median value of gene expression. The HR was calculated based on Cox proportional hazard model. The dotted line indicates 95% CI.

Survival analysis using the GEPIA online tool showed that patients with high expression levels of the *HADH*, *ENO1*, and *FH* genes had lower survival percent compared with patients with low expression levels (*HADH*: HR = 2.2, *p* = 0.0054; *ENO1*: HR = 2.1, *p* = 0.0083; and *FH*: HR = 1.8, *p* = 0.034; Figure 4). The other three genes *GLUL*, *IDH2*, and *LTF* did not correlate with AML prognosis (Figure 4).

## 4 Discussion

The identification of molecular markers, including protein and gene expression and mutations, provide an additional reference for predicting treatment responses or prognosis of patients with heterogeneity AMLs [9,12,13,14,15,16]. Our study identified that 138 proteins were differentially expressed in the serum of patients with favorable-risk, intermediate-risk, and poor-risk AMLs. Proteins, including PSMD1, HADH, ENO1, IDH2, LTF, RPSA, RPS20, and GLUL, were associated with AML development by involving a variety of biological processes related to cell interaction, cellular responses, RNA processing, amino acids biosynthesis, and metabolism.

Proteins including IDH2, GLUL, LTF, and HADH were upregulated in patients with poor-risk AMLs compared with patients with favorable-risk AMLs. Mutations in the *IDH2* gene have been identified in approximately 10% of AML patients [24,25], and there is a controversy over its association with AML prognosis [24,25,26,27]. *IDH2* mutation leads to an elevated level of serum 2-hydroxyglutarate and induces DNA hypermethylation and the subsequent stagnation of cellular differentiation [28]. Also, a high level of 2-hydroxyglutarate is significantly associated with poor OS and disease-free survival in patients with AMLs [29]. Accordingly, *IDH2* mutation inhibitors, including enasidenib, are proposed for the treatment of AML with *IDH2* mutations [30,31,32]. Our TMT-labeling proteomics analysis and ELISA assay confirmed that the level of IDH2 protein was upregulated in patients with poor-risk AMLs compared with favorable-risk AMLs, irrespective of genetic abnormalities. It was insignificantly associated with AML prognosis (*p* = 0.055). Further validation experiments using larger cohorts might confirm the association of its upregulation with the prognosis in AML [33].

The FH enzyme catalyzes the reversible hydration of fumarate to malate in the tricarboxylic acid (TCA) cycle. FH functions as a tumor suppressor in lyomeioma and renal kidney cancer [34]. FH mutation results in the accumulation of fumarate and is associated with human cancers, including hereditary leiomyomatosis and renal cell cancer [35,36,37,38,39]. GLUL catalyzes ammonia ligation and converts glutamate to glutamine. The levels of GLUL, glutamate, and glutamine in AML patients have not been reported. Elevated glutamate is correlated with a high level of oncometabolite 2-hydroxyglutarate in AML patients with *IDH*1/2 mutation [40]. Also, the mutation in *GLUL* was related to brain malformations in neonates [41]. *GLUL* deficiency reduced the level of systemic glutamine and subsequent multi-organ failures or death [42]. ENO1 is a key glycolytic enzyme and is upregulated in multiple human cancers, including ovarian cancer [43], hepatocellular carcinoma [44], and gastric cancer [45]. In addition, the potent tumor suppressor role of LTF has been shown in hepatic steatosis [20] and several human cancers, including nasopharyngeal carcinoma [46], prostate cancer [47], and clear cell renal cell carcinoma [48]. Nevertheless, there is no information reporting the association of the dysregulation and mutations of these proteins/genes with AML prognosis or responses to treatment. Our present study initially identified the elevated expression of FH, GLUL, ENO1, and LTF, in patients with poor-risk AMLs compared with patients who had intermediate/favorable-risk AMLs. Also, the ELISA assay showed that high levels of GLUL (*p* = 0.039), ENO1 (*p* = 0.053), LTF (*p* = 0.016), and FH (*p* = 0.059) proteins were associated with poor prognosis in AML patients. Also, online verification using the GEPIA tool confirmed the significant associations of *FH* and *ENO1* gene expression with poor prognosis in AML patients, showing the potential association of them with AML development.

The PPI network and module analysis identified a cluster of DEPs associated with ribosomes. Fourteen of the 15 DEPs in module 1, including RPSA, RPS6, RPS20, and RPL5, were upregulated in poor-risk AMLs compared with favorable-risk AMLs. RPSA, RPS6, RPS20, and RPL5 proteins were associated with “GO:0006413: translational initiation,” “GO:0006364: rRNA processing,” “GO:0019083: viral transcription,” and “hsa03010: Ribosome.” Also, other DEPs in module 1, including PSMD1, PSMD12, PSMC4, CCT8, and CCT4, were associated with biological processes related to TNF-mediated signaling pathways, cellular amino acid metabolism, and toxin transport. RPL5 is a tumor suppressor [49]. The missense mutation of the *RPL5* gene might be correlated with *TP53* ^mut^ [50]. A low level of *RPL5* expression in multiple myeloma patients was correlated with a higher relapse rate following bortezomib treatment [51]. PSMD1, PSMD12, and PSMC4 are three core components of the proteasome. A mutation in PSMD12 has been identified in a multiplex family with intellectual disability [52]. However, the association of these genes or proteins with AML has not been reported yet. The differential expression levels of these proteins between patients with poor-risk and favorable-risk AMLs showed that these factors might be associated with the development of AML.

This study included two limitations: the lack of a control group for the MS/MS study and the inconsistency between the validation and experiment cohorts. The first limitation prevented the comparison of proteomic differences between healthy individuals and AML patients However, six proteins (including GLUL, FH, LTF, ENO1, HADH, and IDH2) with relative high FC ratios between patients with poor-risk, favorable-risk, and intermediate-risk AMLs were identified as potent prognostic proteins in AML. The inconsistency between the validation and experiment cohorts might affect the ELISA results, but the identification of common DEPs might indicate that the research results are reliable.

## 5 Conclusion

In summary, this present study highlighted a cluster of proteins that have potent prognostic impacts on AML. Proteins including FH, ENO1, IDH2, GLUL, LTF, HADH, and RPL5 that were upregulated in patients with poor-risk AMLs as compared with favorable/intermediate-risk AMLs were associated with TCA, glycolysis, and rRNA processing. The high expression levels of these proteins might increase the risk of poor prognosis in AML patients. Further research should focus on research trials with large cohorts to validate the association of these potent biomarkers with AML prognosis.

## Supplementary Material

Supplementary material
