# Supplementary material for: TMT-based comprehensive proteomic profiling identifies serum prognostic signatures of acute myeloid leukemia
Source: Open Med (Wars). 2023 Mar 30;18(1):20220602. doi: 10.1515/med-2022-0602 (PMC10066874; doi:10.1515/med-2022-0602)
Supplement: Supplementary material [file med-2022-0602-sm.pdf]

Supplementary material

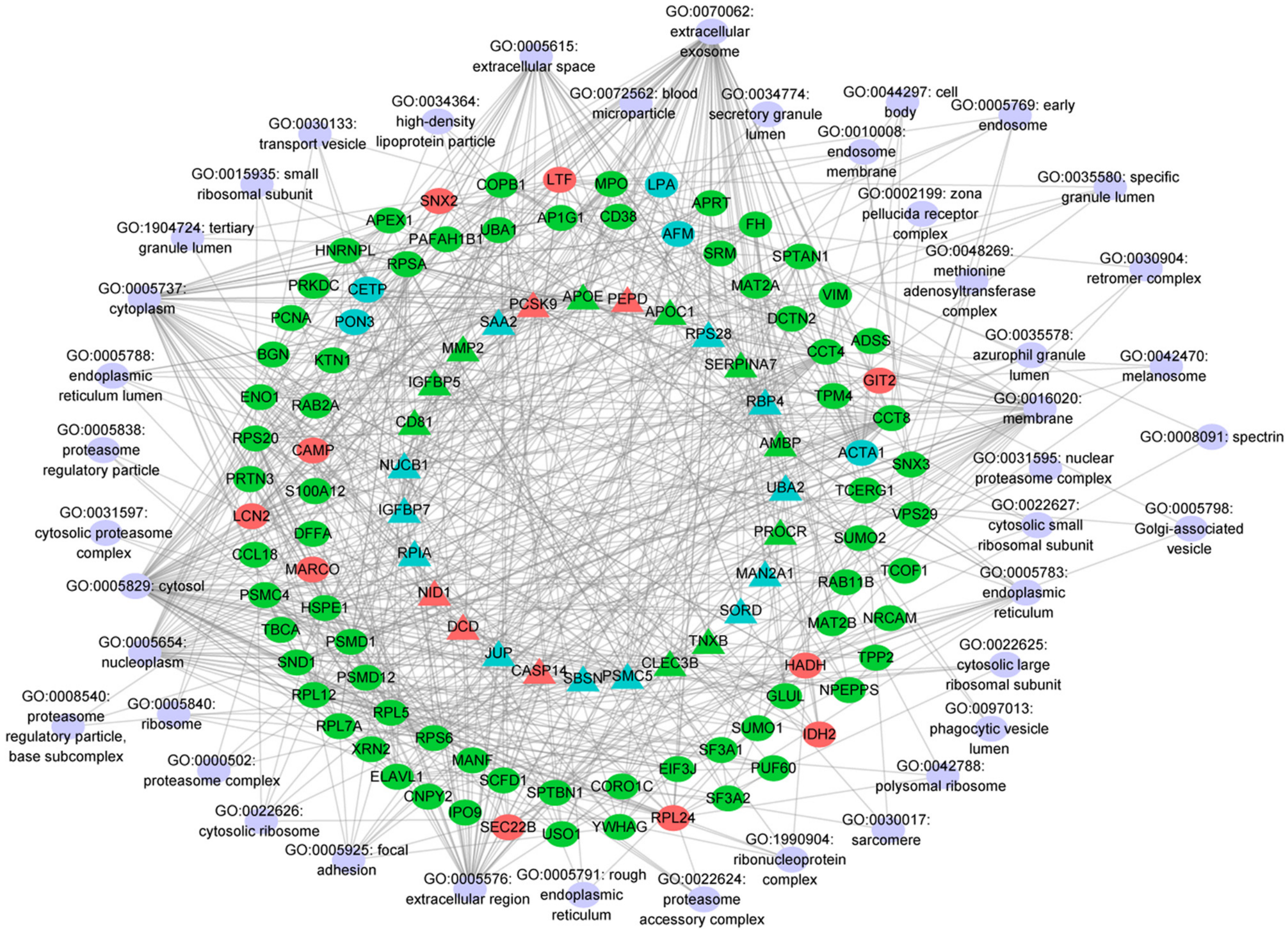

Figure S1: The protein-protein interaction (PPI) network of differentially expressed proteins (DEPs) in patients with acute myeloid leukemia, involving cellular components. DEPs are indicated by different comparisons: favorable-risk vs. intermediate-risk (Cyan), favorable-risk vs. poor-risk (Red), and intermediate-risk vs. poor-risk (Green), respectively. Upregulated and downregulated proteins are shown by circles and triangles, respectively. Cellular components are noted by blue circles.

Table S1: The demographic characteristics of patients with acute myeloid leukemia included for TMT-labeling analysis

Group	Diagnosis	Gender	Age	WBC	Mutation	Fusion gene	Karyotype	BM blast (%)
Intermediate-risk	M5	Female	54	2.71	(–)	*WT1*	46, XX[20]	NA
	AML-M2b	Male	43	4.16	*WT1*	*AML*-ETP	46, XY	81
	AML-M2b	Male	66	2.05	*WT1*	*AML*-ETO	46, XY	75.5
	AML-M1	Female	62	152.85	(–)	(–)	46, XX[20]	NA
	AML-M5b	Female	45	0.84	*DNMT3A*, *IDH2*/R172, *ASXL1*	(–)	46, XX[20]	90.5
	AML-M5	Female	50	2.43	*DNMT3A*; *ASXL1*	(–)	46, XX[20]	94.5
	AML-M	Female	25	122.13	NA	(–)	46, XX[10]	94.5
	AML-M5	Female	27	52.47	NA	(–)	46, XX[20]	77
	AML-M5	Female	54	91.06	*TET2*	(–)	46, XX, t(5,6)(q31,q21)[9]/ XX[11]	79.5
	AML	Female	10	0.67	*TP53* (–), *IPH2*, *DNMT3A* (missense)	(–)	46, XX[20]	NA
	AML-M4b	Female	55	18.05	*WT1*	(–)	46, XX	NA
	AML-M5	Female	55	11.62	(–)	(–)	47, XX, +8[20]	NA
	AML-M2b	Male	58	18.29	*C-kit*/*D816*, *TET2*, *ASXL1*	(–)	47, XY, t(8;21)(q22;q22), +15[2]/46 idem, -Y[18]	91
	AML-M5	Male	61	0.33	*IDH2*; *DNMT3A*; *ASXL1*	(–)	47, XY, +8[20]	NA
	AML-M6	Male	43	189.37	NA	(–)	46, XY[6]	80
	AML-M5a	Male	51	12.93	*TET2*, *ASXL1*, *WT1*	(–)	46, XY	NA
	AML-M4	Male	26	3.38	*WT1*	(–)	46, XY[18]	75
	AML-M4	Male	40	19.62	(–)	(–)	46, XY, t(2;9) (q31;p22)[20]	87.5
	AML-M5	Female	40	1.62	*WT1*		46, XX[20]	52.5
Favorable-Risk	AML-M5b	Male	50	2.78	*IDH2*, *NPM1*	*WT1*(+)	NA	85
	M2b	Female	45		(–)	*AML*-ETO(+)	46, XX, t(8;21) (q22;q22)[19]	NA
	AML-M2	Male	43	0.69	*TET2*, *ASXL1*	*AML*-ET0	45, XY, t(8,21)(q22,q22)	65.5
	AML-M4b	Female	78	123	*CEBPA*; *WT1*	(–)	46, XX[20]	78
	AML-M4	Female	32	29.21	Biallelic mutated *CEBPA*	(–)	46, XX[20]	49.5
	AML-M4b	Female	45	12.92	Biallelic mutated *CEBPA*	(–)	46, XX[20]	77.5
	AML-M6	Female	44	2.47	*NPM1*	(–)	46, XX	98.5
	M4	Female	24	2.76	*TP53*(–), *NPM1* (Frameshift)	(–)	46, XX[3]	NA
	AML-M2a	Male	77	65.49	*CEBPA* (TAD1 region), *WT1*	(–)	NA	56
	AML-M5	Male	10	15.12	Biallelic mutated *CEBPA*	(–)	46, XY[20]	74
	AML-M5	Male	69	67.78	*NPM1*	(–)	46, XY	70
	AML-M1	Male	49	19.07	*NPM1*	(–)	NA	NA
	AML-M5b	Male	49	7.16	*NPM1*; *DNMT3A*	(–)	46, XY[20]	NA
	AML-M2b	Male	72	27.76	*WT1*, *NPM1*	(–)	46, XY[10]	86
Poor-risk	AML-M4b	Female	15	9.72	*CEBPA*	*BCR*-ABL	46, XX[20]	61
	AML-M2	Male		46.8	*TP53*(–)		46, XY[20]	NA

(*Continued*)

Table S1: *Continued*

Group	Diagnosis	Gender	Age	WBC	Mutation	Fusion gene	Karyotype	BM blast (%)
						AML-ETO(+)		
	AML-m4?	Female	44	5.19	*C-kit/D816*, *TET2*, *ASXL1*	(–)	Complex	63
	AML-M1	Female	59	337.28	*FLT3*-ITD, *NPM1*, *IDH2*^{R140}, *DNMT3A*, *TET2*, *ASXL1*	(–)	46, XX[6]	94
	AML-M4	Female	50	67.94	*NPM1*(Exon12)	(–)	46, XX, inv(3)(q21q26)[6]	80
	AML-M2	Male	16	31.2	(–)	(–)	42–44, XY, add(3)(q21), add(8)(q22), add(12)(p13), inc(2)	NA
	AML	Male	71	68.83	*ASXL1*(Frameshift), *FLT3*-ITD(+)	(–)	46, XY[20]	NA
	AML-m4?	Male	41	2.44	*CEBPA*(+), *TET2*(+), *ASXL*(+)	(–)	47, XY, +Y[4], 46, XY[2]	NA
	AML-M5	Male	46	70.32	*FLT3*-ITD; *NPM1*	(–)	NA	NA
	AML	Male	12	5.69	*FLT3* (missense)	(–)	47, XY+8[11]	NA
	AML	Male	61	25.94	*TP53*(–), *FLT3*-ITD	(–)	47, XY+1[9]	NA
	AML	Male	63	0.84	*TP53*(+), *ASXL1*	(–)	multiple chromosomal aberrations	NA
	AML-M5b	Female	27	76.64	*WT1*	(–)	46, XX, t(6;11)(q27;q23)[20]	88.5
	AML-M4b	Male	56	59.21	*FLT3*-ITD, *CEBPA*	(–)	—	NA
Poor-risk	AML-M2	Male	61	0.67	(–)	(–)	46, XY, t(11;12)(q11;p11)[20]	79
Poor-risk	AML-M4	Male	50	38.82	(–)	(–)	NA	NA
Poor-risk	AML-M2a	Female	62	7.67	*CEBPA*, *FLT3*, *TET3*	(–)	46, XX[10]	NA
Poor-risk	AML-M4	Female	50	4.21	*CEBPA*, *TET2*	(–)	46, XX[20]	29.50%

ITD, internal tandem duplication; NA, not applicable; (–), negative report.

Table S2: The list of the differentially expressed proteins (DEPs) in patients with acute myeloid leukemia

Protein accession	Protein description	PR/FR Ratio	Regulation	PR/FR P value	MW [kDa]
P02788	Lactotransferrin (LTF)	3.152	Up	3.72E-02	78.181
P49913	Cathelicidin antimicrobial peptide (CAMP)	3.071	Up	2.54E-02	19.301
P80188	Neutrophil gelatinase-associated lipocalin (LCN2)	2.674	Up	4.80E-02	22.588
P48735	Isocitrate dehydrogenase [NADP], mitochondrial (IDH2)	2.252	Up	4.33E-02	50.909
P15104	Glutamine synthetase (GLUL)	2.063	Up	8.70E-03	42.064
O43747	AP-1 complex subunit gamma-1 (AP1G1)	1.861	Up	3.43E-02	91.35
Q16836	Hydroxyacyl-coenzyme A dehydrogenase, mitochondrial (HADH)	1.736	Up	2.57E-02	34.293
O75396	Vesicle-trafficking protein SEC22b (SEC22B)	1.612	Up	4.13E-02	24.593
Q15651	High mobility group nucleosome-binding domain-containing protein 3 (HMGN3)	1.502	Up	2.48E-02	10.666
Q14161	ARF GTPase-activating protein GIT2 (GIT2)	1.502	Up	1.59E-02	84.542
Q86UP2	Kinectin (KTN1)	1.432	Up	4.53E-02	156.27
O60749	Sorting nexin-2 (SNX2)	1.43	Up	3.20E-02	58.47
P83731	60S ribosomal protein L24 (RPL24)	1.409	Up	2.49E-02	17.779
Q9UEW3	Macrophage receptor MARCO (MARCO)	1.288	Up	1.68E-02	52.657
Q16706	Alpha-mannosidase 2 (MAN2A1)	0.831	Down	8.40E-03	131.14
Q9H8L6	Multimerin-2 (MMRN2)	0.828	Down	2.16E-02	104.41
Q9HBW9	Adhesion G protein-coupled receptor L4 (ADGRL4)	0.814	Down	1.53E-02	77.81
Q8NBP7	Proprotein convertase subtilisin/kexin type 9 (PCSK9)	0.812	Down	3.00E-02	74.285
Q9UKY7	Protein CDV3 homolog (CDV3)	0.811	Down	3.51E-02	27.335
P14543	Nidogen-1 (NID1)	0.798	Down	3.20E-02	136.38
P05155	Plasma protease C1 inhibitor (SERPING1)	0.791	Down	4.15E-02	55.154
P12955	Xaa-Pro dipeptidase (PEPD)	0.786	Down	2.38E-02	54.548
P60033	CD81 antigen (CD81)	0.784	Down	1.67E-02	25.809
Q86UD1	Out at first protein homolog (OAF)	0.782	Down	3.37E-02	30.688
Q6YHK3	CD109 antigen (CD109)	0.769	Down	4.37E-02	161.69
O95998	Interleukin-18-binding protein (IL18BP)	0.768	Down	4.19E-02	21.099
P31944	Caspase-14 (CASP14)	0.768	Down	1.17E-02	27.679
Q86SQ4	Adhesion G-protein coupled receptor G6 (ADGRG6)	0.766	Down	7.36E-03	136.69
P81605	Dermcidin (DCD)	0.759	Down	4.70E-03	11.284

Protein accession	Protein description	PR/IR Ratio	Regulation	PR/IR P value	MW [kDa]
Q8N4W9	Zinc finger protein 808 (ZNF808)	8.231	Up	5.75E-05	104.82
Q6P387	Uncharacterized protein C16orf46 (C16orf46)	3.194	Up	5.06E-03	43.417
P80511	Protein S100-A12 (S100A12)	2.071	Up	1.30E-03	10.575
P61604	10 kDa heat shock protein, mitochondrial (HSPE1)	1.958	Up	8.98E-03	10.932
P46777	60S ribosomal protein L5 (RPL5)	1.818	Up	1.26E-02	34.362
P05164	Myeloperoxidase (MPO)	1.814	Up	2.05E-02	83.868

(*Continued*)

Table S2: *Continued*

Protein accession	Protein description	PR/IR Ratio	Regulation	PR/IR P value	MW [kDa]
P27695	DNA-(apurinic or apyrimidinic site) lyase (APEX1)	1.78	Up	4.78E-02	35.554
O00273	DNA fragmentation factor subunit alpha (DFFA)	1.754	Up	2.70E-02	36.521
P63165	Small ubiquitin-related modifier 1 (SUMO1)	1.739	Up	8.50E-03	11.557
O43747	AP-1 complex subunit gamma-1 (AP1G1)	1.71	Up	4.10E-02	91.35
Q9UHX1	Poly(U)-binding-splicing factor PUF60 (PUF60)	1.685	Up	1.58E-03	59.875
P61019	Ras-related protein Rab-2A (RAB2A)	1.672	Up	1.74E-02	23.545
P15104	Glutamine synthetase (GLUL)	1.669	Up	3.99E-02	42.064
P24158	Myeloblastin (PRTN3)	1.668	Up	3.47E-02	27.807
Q9H0D6	5'-3' exoribonuclease 2 (XRN2)	1.656	Up	2.87E-02	108.58
Q8WVM8	Sec1 family domain-containing protein 1 (SCFD1)	1.649	Up	3.92E-03	72.379
O14776	Transcription elongation regulator 1 (TCERG1)	1.641	Up	5.53E-03	123.9
Q13428	Treacle protein (TCOF1)	1.618	Up	1.52E-02	152.1
P08670	Vimentin (VIM)	1.6	Up	1.84E-02	53.651
P55145	Mesencephalic astrocyte-derived neurotrophic factor (MANF)	1.596	Up	2.30E-02	20.7
P29144	Tripeptidyl-peptidase 2 (TPP2)	1.58	Up	8.78E-03	138.35
P62753	40S ribosomal protein S6 (RPS6)	1.579	Up	3.16E-02	28.68
P78527	DNA-dependent protein kinase catalytic subunit (PRKDC)	1.568	Up	1.27E-02	469.08
Q13813	Spectrin alpha chain, non-erythrocytic 1 (SPTAN1)	1.563	Up	4.08E-02	284.54
Q15428	Splicing factor 3A subunit 2 (SF3A2	1.562	Up	4.12E-02	49.255
P14866	Heterogeneous nuclear ribonucleoprotein L (HNRNPL	1.545	Up	2.15E-02	64.132
Q96P70	Importin-9 (IPO9)	1.493	Up	1.48E-02	115.96
Q9Y2B0	Protein canopy homolog 2 (CNPY2)	1.47	Up	1.41E-02	20.652
P61956	Small ubiquitin-related modifier 2 (SUMO2)	1.461	Up	2.43E-02	10.871
P67936	Tropomyosin alpha-4 chain (TPM4)	1.459	Up	4.72E-02	28.521
P30050	60S ribosomal protein L12 (RPL12)	1.456	Up	6.86E-03	17.818
Q01082	Spectrin beta chain, non-erythrocytic 1 (SPTBN1)	1.448	Up	1.04E-02	274.61
Q86UP2	Kinectin (KTN1)	1.437	Up	9.72E-03	156.27
P53618	Coatomer subunit beta (COPB1)	1.437	Up	2.69E-02	107.14
P36543	V-type proton ATPase subunit E 1 (ATP6V1E1)	1.43	Up	2.11E-02	26.145
Q15717	ELAV-like protein 1 (ELAVL1)	1.418	Up	1.03E-02	36.091
Q15459	Splicing factor 3A subunit 1 (SF3A1)	1.408	Up	3.00E-02	88.885
P55774	C-C motif chemokine 18 (CCL18)	1.403	Up	3.95E-02	9.8487
O60763	General vesicular transport factor p115 (USO1)	1.4	Up	3.66E-02	107.89
Q7KZF4	Staphylococcal nuclease domain-containing protein 1 (SND1)	1.394	Up	3.35E-02	102
P0DP08	Immunoglobulin heavy variable 4-38-2 (IGHV4-38-2)	1.392	Up	4.00E-02	13.016
Q9UBQ0	Vacuolar protein sorting-associated protein 29 (VPS29)	1.39	Up	2.80E-02	20.505
P43686	26S proteasome regulatory subunit 6B (PSMC4)	1.39	Up	2.25E-02	47.366
O60493	Sorting nexin-3 (SNX3)	1.384	Up	3.49E-02	18.762
P28907	ADP-ribosyl cyclase/cyclic ADP-ribose hydrolase 1 (CD38)	1.364	Up	3.02E-02	34.328

(*Continued*)

Table S2: *Continued*

Protein accession	Protein description	PR/IR Ratio	Regulation	PR/IR P value	MW [kDa]
P08865	40S ribosomal protein SA (RPSA)	1.343	Up	4.16E-02	32.854
Q15907	Ras-related protein Rab-11B (RAB11B)	1.343	Up	2.48E-02	24.488
P43034	Platelet-activating factor acetylhydrolase IB subunit alpha (PAFAH1B1)	1.335	Up	6.30E-03	46.637
P30419	Glycylpeptide N-tetradecanoyltransferase 1 (NMT1)	1.329	Up	1.19E-02	56.806
P07954	Fumarate hydratase, mitochondrial (FH)	1.326	Up	1.71E-02	54.636
P19623	Spermidine synthase (SRM)	1.323	Up	2.92E-02	33.824
O75351	Vacuolar protein sorting-associated protein 4B (VPS4B)	1.318	Up	3.06E-02	49.301
O00232	26S proteasome non-ATPase regulatory subunit 12 (PSMD12)	1.311	Up	6.44E-03	52.904
Q92823	Neuronal cell adhesion molecule (NRCAM)	1.31	Up	2.87E-02	143.89
Q9BRA2	Thioredoxin domain-containing protein 17 (TXNDC17)	1.309	Up	4.48E-02	13.941
P31153	S-adenosylmethionine synthase isoform type-2 (MAT2A)	1.305	Up	3.87E-02	43.66
O75822	Eukaryotic translation initiation factor 3 subunit J (EIF3J)	1.298	Up	2.04E-02	29.062
P06733	Alpha-enolase (ENO1)	1.288	Up	4.93E-02	47.168
P12004	Proliferating cell nuclear antigen (PCNA)	1.279	Up	2.37E-02	28.768
Q9ULV4	Coronin-1C (CORO1C)	1.271	Up	1.04E-02	53.248
P50991	T-complex protein 1 subunit delta (CCT4)	1.271	Up	4.05E-02	57.924
P22314	Ubiquitin-like modifier-activating enzyme 1 (UBA1)	1.265	Up	3.73E-02	117.85
O75347	Tubulin-specific chaperone A (TBCA)	1.261	Up	4.25E-02	12.855
P30520	Adenylosuccinate synthetase isozyme 2 (ADSS)	1.249	Up	4.54E-02	50.097
P60866	40S ribosomal protein S20 (RPS20)	1.248	Up	2.00E-02	13.373
P07741	Adenine phosphoribosyltransferase (APRT)	1.237	Up	2.03E-02	19.608
P62424	60S ribosomal protein L7a (RPL7A)	1.233	Up	4.51E-02	29.995
Q13561	Dynactin subunit 2 (DCTN2)	1.233	Up	1.96E-02	44.23
Q9NZL9	Methionine adenosyltransferase 2 subunit beta (MAT2B)	1.231	Up	1.64E-02	37.551
P09417	Dihydropteridine reductase (QDPR)	1.226	Up	2.52E-02	25.789
Q99460	26S proteasome non-ATPase regulatory subunit 1 (PSMD1)	1.223	Up	4.76E-02	105.84
P50990	T-complex protein 1 subunit theta (CCT8)	1.22	Up	4.72E-02	59.62
P55786	Puromycin-sensitive aminopeptidase (NPEPPS)	1.219	Up	2.38E-02	103.28
P21810	Biglycan (BGN)	1.216	Up	7.22E-03	41.654
O43598	2'-deoxynucleoside 5'-phosphate N-hydrolase 1 (DNPH1)	1.206	Up	3.06E-02	19.108
P61981	14-3-3 protein gamma (YWHAG)	1.201	Up	2.56E-02	28.302
P05452	Tetranectin (CLEC3B)	0.833	Down	7.06E-03	22.537
P26639	Threonine–tRNA ligase, cytoplasmic (TARS)	0.832	Down	1.54E-02	83.434
Q9ULI3	Protein HEG homolog 1 (HEG1)	0.832	Down	2.63E-02	147.46
Q9UNN8	Endothelial protein C receptor (PROCR)	0.828	Down	4.17E-02	26.671
P60033	CD81 antigen (CD81)	0.824	Down	2.94E-02	25.809
P05543	Thyroxine-binding globulin (SERPINA7)	0.81	Down	8.82E-03	46.324
Q9BWP8	Collectin-11 (COLEC11)	0.81	Down	4.37E-02	28.665

(*Continued*)

Table S2: *Continued*

Protein accession	Protein description	PR/IR Ratio	Regulation	PR/IR P value	MW [kDa]
P05155	Plasma protease C1 inhibitor (SERPING1)	0.809	Down	4.82E-02	55.154
P02753	Retinol-binding protein 4 (RBP4)	0.801	Down	3.39E-02	23.01
P24593	Insulin-like growth factor-binding protein 5 (IGFBP5)	0.791	Down	1.04E-02	30.57
P22105	Tenascin-X (TNXB)	0.788	Down	2.73E-02	458.22
Q9H8L6	Multimerin-2 (MMRN2)	0.788	Down	2.63E-02	104.41
Q8NCW5	NAD(P)H-hydrate epimerase (NAXE)	0.786	Down	2.26E-02	31.674
Q86SQ4	Adhesion G-protein coupled receptor G6 (ADGRG6)	0.779	Down	2.21E-02	136.69
P02649	Apolipoprotein E (APOE)	0.778	Down	3.00E-03	36.154
P08253	72 kDa type IV collagenase (MMP2)	0.776	Down	3.03E-02	73.881
O95998	Interleukin-18-binding protein (IL18BP)	0.775	Down	3.45E-02	21.099
P02760	Protein AMBP (AMBP)	0.774	Down	1.31E-02	38.999
P13598	Intercellular adhesion molecule 2 (ICAM2)	0.759	Down	7.38E-03	30.654
P02654	Apolipoprotein C-I (APOC1)	0.757	Down	4.41E-02	9.3318

Protein accession	Protein description	IR /FR Ratio	Regulation	IR/FR P value	MW [kDa]
P05451	Lithostathine-1-alpha (REG1A)	1.548	Up	4.54E-02	18.731
P02753	Retinol-binding protein 4 (RBP4)	1.291	Up	1.76E-03	23.01
Q15166	Serum paraoxonase/lactonase 3 (PON3)	1.548	Up	3.63E-02	39.607
Q8NCW5	NAD(P)H-hydrate epimerase (NAXE)	1.278	Up	1.55E-02	31.674
P01859	Immunoglobulin heavy constant gamma 2 (IGHG2)	1.259	Up	3.41E-02	35.9
Q9NS71	Gastrokine-1 (GKN1)	1.57	Up	4.04E-03	21.999
Q9NQ79	Cartilage acidic protein 1 (CRTAC1)	1.313	Up	2.02E-03	71.42
P11597	Cholesteryl ester transfer protein (CETP)	1.326	Up	3.94E-03	54.756
P43652	Afamin (AFM)	1.46	Up	2.95E-02	69.068
P68133	Actin, alpha skeletal muscle (ACTA1)	1.415	Up	2.70E-02	42.051
Q9UBT2	SUMO-activating enzyme subunit 2 (UBA2)	0.676	Down	2.03E-02	71.223
Q00796	Sorbitol dehydrogenase (SORD)	0.651	Down	3.67E-02	38.324
P14151	L-selectin (SELL)	0.827	Down	4.30E-02	42.187
Q6UWP8	Suprabasin (SBSN)	0.686	Down	5.20E-04	60.54
P0DJI9	Serum amyloid A-2 protein (SAA2)	0.48	Down	1.59E-02	13.527
P62857	40S ribosomal protein S28 (RPS28)	0.649	Down	2.56E-02	7.8409
P49247	Ribose-5-phosphate isomerase (RPIA)	0.568	Down	4.39E-02	33.269
P62195	26S proteasome regulatory subunit 8 (PSMC5)	0.732	Down	3.38E-03	45.626
Q02818	Nucleobindin-1 (NUCB1)	0.689	Down	4.77E-02	53.879
Q16706	Alpha-mannosidase 2 (MAN2A1)	0.806	Down	4.28E-02	131.14
P08519	Apolipoprotein(a) (LPA)	0.722	Down	1.79E-02	501.31
P14923	Junction plakoglobin (JUP)	0.637	Down	2.94E-02	81.744
Q16270	Insulin-like growth factor-binding protein 7 (IGFBP7)	0.782	Down	4.56E-02	29.13

FR, favorable-risk; IR, intermediate-risk; PR, poor-risk.

Table S3: The Gene Ontology functional terms and Kyoto Encyclopedia of Genes and Genomes (KEGG) pathways associated with the differentially expressed proteins by different comparisons

Term	Count	Genes	P Value
**PR/FR -Gene Ontology Biological process**			
GO:0006508:proteolysis	5	CASP14, DCD, ADGRG6, PEPD, LTF	2.75E-03
GO:0050829:defense response to Gram-negative bacterium	3	DCD, CAMP, LTF	7.02E-03
GO:0045087:innate immune response	5	MARCO, LCN2, SERPING1, CAMP, LTF	7.23E-03
GO:0072675:osteoclast fusion	2	CD81, CD109	8.12E-03
GO:0061844:antimicrobial humoral immune response mediated by antimicrobial peptide	3	DCD, CAMP, LTF	9.54E-03
GO:0050830:defense response to Gram-positive bacterium	3	DCD, CAMP, LTF	1.06E-02
**PR/FR -Gene Ontology Molecular Function**			
GO:1990459:transferrin receptor binding	2	SNX2, CD81	1.51E-02
GO:0043236:laminin binding	2	ADGRG6, NID1	3.54E-02
GO:0001530:lipopolysaccharide binding	2	CAMP, LTF	4.74E-02
**PR/FR -Gene Ontology Cellular Component**			
GO:0070062:extracellular exosome	14	DCD, CD81, IDH2, PEPD, NID1, IL18BP, MAN2A1, MMRN2, LCN2, SERPING1, RPL24, GLUL, CAMP, LTF	1.50E-06
GO:0005615:extracellular space	9	DCD, CD109, MMRN2, LCN2, SERPING1, PCSK9, CAMP, IL18BP, LTF	2.70E-03
GO:0035580:specific granule lumen	3	LCN2, CAMP, LTF	3.11E-03
GO:0005576:extracellular region	9	DCD, CD109, LCN2, SERPING1, PCSK9, NID1, CAMP, IL18BP, LTF	5.18E-03
GO:0042581:specific granule	2	CAMP, LTF	1.73E-02
GO:0009986:cell surface	4	CD109, ADGRG6, PCSK9, LTF	5.17E-02
**PR/IR -Gene Ontology Biological processes**			
GO:0002181:cytoplasmic translation	6	RPL7A, RPL5, RPL12, RPS6, RPS20, RPSA	6.55E-05
GO:0006397:mRNA processing	6	HNRNPL, TCERG1, SF3A1, SF3A2, PUF60, XRN2	3.29E-03
GO:0006412:translation	6	RPL7A, RPL5, RPL12, RPS6, RPS20, RPSA	4.43E-03
GO:0010977:negative regulation of neuron projection development	4	CD38, VIM, APOE, PAFAH1B1	4.84E-03
GO:0045727:positive regulation of translation	4	RPL5, PRKDC, VIM, ELAVL1	6.98E-03
GO:0030036:actin cytoskeleton organization	5	TNXB, SPTAN1, SPTBN1, CORO1C, PAFAH1B1	1.15E-02
GO:0006641:triglyceride metabolic process	3	TNXB, APOC1, APOE	1.29E-02
GO:0007568:aging	5	IGFBP5, APEX1, MMP2, SERPING1, MPO	1.33E-02
GO:1903241:U2-type prespliceosome assembly	2	SF3A1, SF3A2	1.43E-02
GO:0034447:very-low-density lipoprotein particle clearance	2	APOC1, APOE	1.43E-02
GO:0061077:chaperone-mediated protein folding	3	DFFA, CCT8, CCT4	1.66E-02
GO:1901998:toxin transport	3	SCFD1, CCT8, CCT4	1.89E-02
GO:0006556:S-adenosylmethionine biosynthetic process	2	MAT2A, MAT2B	1.91E-02
GO:0006508:proteolysis	7	NPEPPS, COLEC11, PSMC4, MMP2, ADGRG6, TPP2, PRTN3	1.93E-02
GO:0001649:osteoblast differentiation	4	IGFBP5, TPM4, HSPE1, SND1	1.97E-02

(*Continued*)

Table S3: *Continued*

Term	Count	Genes	P Value
GO:0032024:positive regulation of insulin secretion	3	RBP4, CD38, GLUL	1.98E-02
GO:0007507:heart development	5	RBP4, PCNA, PRKDC, HEG1, MMP2	2.06E-02
GO:0006888:ER to Golgi vesicle-mediated transport	4	RAB2A, SCFD1, COPB1, USO1	2.19E-02
GO:0034382:chylomicron remnant clearance	2	APOC1, APOE	2.38E-02
GO:0032526:response to retinoic acid	3	RBP4, MMP2, CD38	2.59E-02
GO:0032456:endocytic recycling	3	SNX3, VPS29, RAB11B	2.78E-02
GO:0000389:mRNA 3'-splice site recognition	2	SF3A1, SF3A2	2.85E-02
GO:0010756:positive regulation of plasminogen activation	2	CLEC3B, ENO1	3.78E-02
GO:0010873:positive regulation of cholesterol esterification	2	APOC1, APOE	4.24E-02
GO:0006457:protein folding	4	CCT8, TBCA, HSPE1, CCT4	4.45E-02
GO:1904851:positive regulation of establishment of protein localization to telomere	2	CCT8, CCT4	4.70E-02
GO:0070301:cellular response to hydrogen peroxide	3	PCNA, APEX1, IL18BP	4.81E-02
**PR/IR-Gene Ontology Molecular Function**			
GO:0003723:RNA binding	29	TCERG1, RPL5, PRKDC, RPL12, USO1, ENO1, ELAVL1, RPL7A, TCOF1, PUF60, SUMO1, SUMO2, YWHAG, SPTBN1, CCT4, SF3A1, SF3A2, RPS6, RPSA, TBCA, HSPE1, SND1, MANF, KTN1, HNRNPL, APEX1, XRN2, RPS20, UBA1	1.48E-10
GO:0005515:protein binding	83	TCERG1, RPL5, VPS29, CD81, ENO1, MPO, ELAVL1, DNPH1, TXNDC17, RPL7A, IPO9, SUMO1, AP1G1, SUMO2, PSMD1, NRCAM, ATP6V1E1, SPTAN1, GLUL, YWHAG, RAB2A, DFFA, TPM4, IGFBP5, HEG1, MMP2, RPS6, BGN, RPSA, CNPY2, SND1, APRT, PROCR, RBP4, XRN2, MMRN2, SERPING1, PRTN3, PAFAH1B1, COLEC11, PSMD12, FH, TNXB, PCNA, DCTN2, NAXE, PRKDC, COPB1, VPS4B, RPL12, USO1, MAT2B, CORO1C, SRM, SNX3, SCFD1, TCOF1, PUF60, MAT2A, S100A12, CCT8, APOE, CCL18, SPTBN1, CCT4, SF3A1, SF3A2, AMBP, TBCA, HSPE1, RAB11B, KTN1, MANF, HNRNPL, PSMC4, APEX1, APOC1, EIF3J, NMT1, TPP2, UBA1, RPS20, VIM	5.70E-07
GO:0045296:cadherin binding	10	RPL7A, PUF60, USO1, CCT8, ENO1, SPTAN1, SND1, SPTBN1, KTN1, RAB11B	2.48E-05
GO:0042802:identical protein binding	20	TCERG1, PCNA, TPM4, DCTN2, NAXE, VPS4B, DNPH1, SRM, QDPR, PUF60, MAT2A, XRN2, EIF3J, TPP2, CD38, VIM, APOE, GLUL, YWHAG, PAFAH1B1	4.84E-04
GO:0003735:structural constituent of ribosome	6	RPL7A, RPL5, RPL12, RPS6, RPS20, RPSA	2.79E-03
GO:0008201:heparin binding	5	CLEC3B, TNXB, APOE, MPO, PAFAH1B1	1.02E-02
GO:0044877:macromolecular complex binding	7	SCFD1, RBP4, PCNA, VPS4B, APEX1, APOE, SPTBN1	1.58E-02

(*Continued*)

Table S3: *Continued*

Term	Count	Genes	P Value
GO:0044183:protein binding involved in protein folding	3	DFFA, CCT8, CCT4	1.88E-02
GO:0060228:phosphatidylcholine-sterol O-acyltransferase activator activity	2	APOC1, APOE	2.91E-02
GO:1990948:ubiquitin ligase inhibitor activity	2	RPL5, RPS20	4.33E-02
**PR/IR-Gene Ontology Cellular Component**			
GO:0070062:extracellular exosome	49	RPL5, CD81, ENO1, MPO, DNPH1, SERPINA7, TXNDC17, IL18BP, NPEPPS, CD38, ATP6V1E1, SPTAN1, GLUL, YWHAG, RAB2A, TPM4, BGN, RPSA, SND1, APRT, PROCR, RBP4, MMRN2, SERPING1, PRTN3, PAFAH1B1, PSMD12, FH, TNXB, PCNA, DCTN2, NAXE, VPS4B, RPL12, MAT2B, SNX3, CLEC3B, CCT8, APOE, SPTBN1, CCT4, AMBP, HSPE1, RAB11B, QDPR, HNRNPL, UBA1, RPS20, VIM	2.75E-22
GO:0005829:cytosol	55	RPL5, VPS29, ENO1, ELAVL1, DNPH1, TXNDC17, NPEPPS, RPL7A, IPO9, SUMO1, AP1G1, PSMD1, ATP6V1E1, SPTAN1, GLUL, YWHAG, RAB2A, DFFA, TPM4, RPS6, RPSA, SND1, APRT, PRTN3, PAFAH1B1, PSMD12, FH, DCTN2, PRKDC, COPB1, VPS4B, RPL12, USO1, MAT2B, SRM, SNX3, SCFD1, TCOF1, MAT2A, S100A12, CCT8, SPTBN1, CCT4, TBCA, RAB11B, MANF, QDPR, PSMC4, EIF3J, NMT1, C16ORF46, TPP2, UBA1, RPS20, VIM	1.81E-10
GO:0016020:membrane	29	RPL5, PSMD12, DCTN2, CD81, PRKDC, COPB1, RPL12, USO1, ICAM2, ENO1, ELAVL1, RPL7A, IPO9, AP1G1, PSMD1, CD38, APOE, SPTAN1, YWHAG, TPM4, RPS6, RPSA, HSPE1, SND1, KTN1, HNRNPL, PSMC4, XRN2, RPS20	5.45E-06
GO:0005576:extracellular region	26	COLEC11, PSMD12, TNXB, NAXE, MPO, SERPINA7, IL18BP, CLEC3B, S100A12, PSMD1, NRCAM, CCT8, APOE, SPTAN1, AMBP, IGFBP5, HEG1, MMP2, BGN, MANF, APRT, PROCR, RBP4, APOC1, SERPING1, PRTN3	7.20E-06
GO:0022626:cytosolic ribosome	6	RPL7A, RPL5, RPL12, RPS6, RPS20, RPSA	2.85E-05
GO:0005615:extracellular space	21	COLEC11, TNXB, IGFBP5, AMBP, NAXE, MMP2, BGN, ENO1, MPO, SERPINA7, MANF, IL18BP, PROCR, CLEC3B, RBP4, MMRN2, SERPING1, S100A12, PRTN3, APOE, CCL18	3.66E-04
GO:0005925:focal adhesion	9	PROCR, RPL7A, RPL5, TPM4, CD81, RPL12, VIM, YWHAG, CORO1C	7.44E-04
GO:0022624:proteasome accessory complex	3	PSMD12, PSMC4, PSMD1	2.74E-03
GO:0044297:cell body	4	NAXE, RPS6, CCT8, CCT4	6.47E-03

(*Continued*)

Table S3: *Continued*

Term	Count	Genes	P Value
GO:1990904:ribonucleoprotein complex	5	HNRNPL, RPL5, RPS6, VIM, ELAVL1	6.74E-03
GO:0015935:small ribosomal subunit	3	RPS6, RPS20, RPSA	7.88E-03
GO:0035578:azurophil granule lumen	4	PSMD1, PRTN3, CCT8, MPO	8.61E-03
GO:0005813:centrosome	8	PROCR, PCNA, DCTN2, VPS4B, APEX1, CCT8, PAFAH1B1, CCT4	1.24E-02
GO:0005654:nucleoplasm	28	TCERG1, RPL5, PSMD12, PCNA, PRKDC, MPO, ELAVL1, TCOF1, PUF60, SUMO1, SUMO2, PSMD1, CCT8, CCT4, DFFA, SF3A1, SF3A2, RPS6, RPSA, APRT, HNRNPL, PSMC4, APEX1, XRN2, C16ORF46, TPP2, RPS20, UBA1	1.33E-02
GO:0048269:methionine adenosyltransferase complex	2	MAT2A, MAT2B	1.38E-02
GO:0034774:secretory granule lumen	4	PSMD12, S100A12, CCT8, APRT	1.62E-02
GO:0005634:nucleus	38	TCERG1, RPL5, FH, PCNA, PRKDC, VPS4B, MAT2B, ENO1, MPO, ELAVL1, DNPH1, ZNF808, NPEPPS, RPL7A, PUF60, SUMO1, SUMO2, S100A12, PSMD1, CD38, APOE, GLUL, SPTBN1, RAB2A, DFFA, SF3A1, SF3A2, MMP2, RPS6, RPSA, SND1, MANF, HNRNPL, PSMC4, APEX1, XRN2, UBA1, PAFAH1B1	1.64E-02
GO:0022627:cytosolic small ribosomal subunit	3	RPS6, RPS20, RPSA	1.83E-02
GO:0010008:endosome membrane	5	SNX3, VPS29, VPS4B, UBA1, CORO1C	2.68E-02
GO:0000502:proteasome complex	3	PSMD12, PSMC4, PSMD1	2.85E-02
GO:0022625:cytosolic large ribosomal subunit	3	RPL7A, RPL5, RPL12	3.04E-02
GO:0002199:zona pellucida receptor complex	2	CCT8, CCT4	3.63E-02
GO:0005938:cell cortex	4	ENO1, SPTAN1, CORO1C, PAFAH1B1	4.03E-02
GO:0008540:proteasome regulatory particle, base subcomplex	2	PSMC4, PSMD1	4.08E-02
GO:0008091:spectrin	2	SPTAN1, SPTBN1	4.08E-02
GO:0005838:proteasome regulatory particle	2	PSMD12, PSMD1	4.52E-02
GO:0005832:chaperonin-containing T-complex	2	CCT8, CCT4	4.96E-02
**PR/IR-KEGG pathways**			
hsa03010:Ribosome	6	RPL7A, RPL5, RPL12, RPS6, RPS20, RPSA	5.98E-03
hsa01230:Biosynthesis of amino acids	4	MAT2A, MAT2B, ENO1, GLUL	1.77E-02
hsa05171:Coronavirus disease - COVID-19	6	RPL7A, RPL5, RPL12, RPS6, RPS20, RPSA	2.76E-02
hsa03050:Proteasome	3	PSMD12, PSMC4, PSMD1	4.49E-02
**IR/FR-Gene Ontology Biological processes**			
GO:0006869:lipid transport	3	CETP, NAXE, LPA	3.87E-03
GO:0010874:regulation of cholesterol efflux	2	CETP, NAXE	7.65E-03
**PR/IR-Gene Ontology Molecular Function**			
GO:0030246:carbohydrate binding	5	RPIA, SELL, MAN2A1, REG1A, SORD	6.50E-05
GO:0070492:oligosaccharide binding	2	SELL, REG1A	1.45E-02
**PR/IR-Gene Ontology Cellular Component**			

(*Continued*)

Table S3: *Continued*

Term	Count	Genes	P Value
GO:0070062:extracellular exosome	18	PON3, CETP, JUP, NAXE, REG1A, AFM, SORD, SBSN, ACTA1, PSMC5, RPS28, RBP4, IGHG2, CRTAC1, MAN2A1, SAA2, NUCB1, IGFBP7	7.26E-13
GO:0005615:extracellular space	13	PON3, CETP, NAXE, GKN1, REG1A, AFM, SORD, ACTA1, RBP4, IGHG2, NUCB1, IGFBP7, LPA	1.29E-07
GO:0005576:extracellular region	11	PON3, CETP, RBP4, IGHG2, JUP, NAXE, GKN1, AFM, NUCB1, IGFBP7, LPA	3.12E-05
GO:0072562:blood microparticle	4	ACTA1, PSMC5, IGHG2, AFM	4.97E-04
GO:0034364:high-density lipoprotein particle	2	CETP, SAA2	2.91E-02
GO:0005801:cis-Golgi network	2	MAN2A1, NUCB1	7.17E-02

FR, favorable-risk. IR, intermediate-risk. PR, poor-risk.
